# Corrigendum

**DOI:** 10.1002/ccr3.1026

**Published:** 2017-07-03

Ghode, T. D. (2017), Ultrasound features of acinic cell carcinoma of the tongue: a rare case report. Clin Case Rep, 5: 406–410. https://doi.org/10.1002/ccr3.755

In the article “Ultrasound features of acinic cell carcinoma of the tongue: a rare case report,” the first sentence in the Introduction should be corrected from:

In this study, a rare case is presented in which a tumor was found on the tip of the tongue.

To:

A rare case is presented in which a tumor was found on the tip of the tongue.

We apologize for any inconvenience caused.

